# Biocompatibility and osteoinductive ability of casein phosphopeptide modified polyetheretherketone

**DOI:** 10.3389/fbioe.2023.1100238

**Published:** 2023-02-08

**Authors:** Peng Qiu, Pin Wang, Min Liu, Tao Dai, Min Zheng, Le Feng

**Affiliations:** ^1^ The Affiliated Stomatological Hospital of Southwest Medical University, Luzhou, Sichuan, China; ^2^ Luzhou Key Laboratory of Oral and Maxillofacial Reconstruction and Regeneration, Luzhou, Sichuan, China; ^3^ Institute of Stomatology, Southwest Medical University, Luzhou, Sichuan, China

**Keywords:** polyetheretherketone, casein phosphopeptide, surface modification, cell adhesion, cell proliferation, osteoinductive ability

## Abstract

Polyetheretherketone (PEEK) is a potential implant material for dental application due to its excellent mechanical properties. However, its biological inertness and poor osteoinductive ability limited its clinical application. Based on a lay-by-layer self-assembly technique, here we incorporated casein phosphopeptide (CPP) onto PEEK surface by a simple two-step strategy to address the poor osteoinductive ability of PEEK implants. In this study, the PEEK specimens were positively charged by 3-ammoniumpropyltriethoxysilane (APTES) modification, then the CPP was adsorbed onto the positively charged PEEK surface electrostatically to obtain CPP-modified PEEK (PEEK-CPP) specimens. The surface characterization, layer degradation, biocompatibility and osteoinductive ability of the PEEK-CPP specimens were studied *in vitro*. After CPP modification, the PEEK-CPP specimens had a porous and hydrophilic surface and presented enhanced cell adhesion, proliferation, and osteogenic differentiation of MC3T3-E1 cells. These findings indicated that CPP modification could significantly improve the biocompatibility and osteoinductive ability of PEEK-CPP implants *in vitro*. In a word, CPP modification is a promising strategy for the PEEK implants to achieve osseointegration.

## 1 Introduction

Dental implant surgery is a vital treatment for dentition defects and edentulism jaw. One of the crucial elements of successful dental implant surgery is the effective osseointegration surrounding implants. Despite being the most widely used implant material due to its excellent biocompatibility and osteoinductive properties, titanium still has some inherent problems. Firstly, there are many case reports indicating that titanium can cause metal allergy (Saha et al., 2020; Tawil et al., 2020; Poli et al., 2021; Buonomo et al., 2022). Secondly, the elastic modulus of titanium (approximately 107 GPa) is far higher than that of human cortical bone (approximately 17 GPa), this mismatched elastic modulus could cause severe stress shielding effect around implants, resulting in bone resorption around implants (Najeeb et al., 2015; Wang et al., 2019; Zhan et al., 2020; Liu et al., 2021). Finally, titanium implants always generate peri-implant artefacts during computed tomography (CT) scanning because of the titanium’s radiopacity, influencing dentists’ clinical evaluation of the osseointegration around the implants (Mancini et al., 2021; Gao et al., 2022; Schulze, 2022). Fortunately, polyetheretherketone (PEEK), a special type of thermoplastic engineering plastic, is a potential substitute for titanium because of its excellent mechanical properties. PEEK’s major beneficial property for dental implant application is its low elasticity modulus (approximately 3–4 GPa) which is similar to the human cortical bone. This similarity could avoid the stress shielding effect and prevent bone resorption around implants (Bonnheim et al., 2019; Li et al., 2019). Moreover, the tensile properties of PEEK (80 MPa) are close to those of bone (104–121 MPa), enamel (47.5 MPa) and dentin (104 MPa), making it a suitable implant material (Najeeb et al., 2016), (Yin et al., 2022), (Major et al., 2019). Finally, the natural radiolucency of PEEK can avoid the effect of peri-implant artefacts during Imaging tests (Bonnheim et al., 2019; Li et al., 2019), (Panayotov et al., 2016; Zhang et al., 2019) However, PEEK’s poor osseointegration presents the main obstacle to its use as dental implants. Bare PEEK implants present smooth surface and hydrophobic, which are the disadvantages of osseointegration (Kwon et al., 2018; Liu et al., 2019). Fortunately, implants surface modification with bioactive molecules is the most promising strategy to improve implants’ osteoinductive ability (Hu et al., 2022; Jin et al., 2022).

Casein phosphopeptide (CPP), a mixture of peptides with different molecular weights, is degraded from bovine casein. It has been demonstrated to be calcium ions (Ca^2+^) chelators (Sun et al., 2018; Luo et al., 2020; Wang et al., 2023). As we know, Ca^2+^ are the main component of teeth and bones. Thus, CPP, which typically functions with amorphous calcium phosphate (ACP), has been applied as a carrier for Ca^2+^ to improve remineralization and inhibit demineralization of enamel surfaces and has also been proven to have osteoinductive ability (Cross et al., 2007; Dashper et al., 2016; Dawood et al., 2018; Alawadhi et al., 2020; Sionov et al., 2021; Huang et al., 2022). Therefore, CPP has great potential for research on implant surface modification and bone tissue engineering. However, there have previously been a few investigations on the CPP coating on implants. Thus, we intended to modify PEEK implants with a CPP layer to address its poor osseointegration. Firstly, direct adsorption of a CPP layer on PEEK implants is undoubtedly an easy and convenient modification method, but this simple adsorption is highly likely to cause weak bonding strength between the CPP layer and the PEEK implants. The CPP layer may drop from the implant surface during the implantation process because of the coating’s poor adhesion. Fortunately, the layer-by-layer self-assembly technique is a reliable coating method, which can assure the strong bonding strength between the coating and the substrate (Elizarova and Luckham, 2018; Gojzewski et al., 2020; Kaizer et al., 2020). In recent years, this technique has been widely used in the surface modification of implants and the synthesis of bone tissue engineering scaffolds (Hu et al., 2021; Shaabani et al., 2021; Qiu et al., 2022a). Its mechanisms are electrostatic interaction, hydrogen bonding, covalent bonding, biological recognition and so on (Borges and Mano, 2014). For example, cationic substances and anionic substances could interact electrostatically in a polyelectrolyte solution (Chenite et al., 2001). Thus, CPP will be introduced onto PEEK implant surface based on a layer-by-layer self-assembly technique. Given the complicated processes of covalent bonding of CPP and the possible effect of the chemical groups taking part in the covalent reaction on the CPP’s ability of chelating Ca^2+^, we decided to incorporate CPP to the surface of PEEK implant by electrostatic adsorption, because this method would not change CPP’s chemical structure.

In this study, we firstly covalently grafted PEEK with amino groups, making PEEK surface positively charged. As CPP carries negative charges, it can electrostatically adsorb onto positively charged PEEK surface. Then, the CPP-modified PEEK implants will be characterized and their biocompatibility and osteoinductive capacity will be evaluated by *in vitro* studies.

## 2 Materials and methods

### 2.1 Materials

PEEK (GEHR^®^ PEEK-1000, Germany), sodium borohydride (NaBH_4_, Jinshan Chemical Reagent Co., LTD., Chengdu, China), dimethylsulfoxide (DMSO), 3-ammoniumpropyltriethoxysilane (APTES, Macklin, China), CPP (Solarbio, China), alpha-Minimum Essential Medium (α-MEM, Procell, China), fetal bovine serum (FBS, TIANHANG, China), 4% paraformaldehyde (Biosharp, China), 4′,6′-diamidino-2-phenylindole (DAPI, Solarbio, China), cell counting kit (CCK-8, Dojindo, Japan), actin-tracker green (Beyotime, China), NBT/BCIP ALP color development kit (Sangon Biotech, China), alkaline phosphate activity assay kit (Nanjing Jiancheng Biotechnology, China), bicinchoninic acid (BCA) protein assay kit (BioTeke, China), Alizarin Red S (ARS, Solarbio, China), RNAsimple Total RNA Kit (TIANGEN, China), ReverTra Ace^®^ qPCR RT Master Mix (TOYOBO, Japan), SYBR Green Realtime PCR Master Mix (TOYOBO, Japan).

## 3 Methods

### 3.1 Sample preparation

**SCHEME 1:**
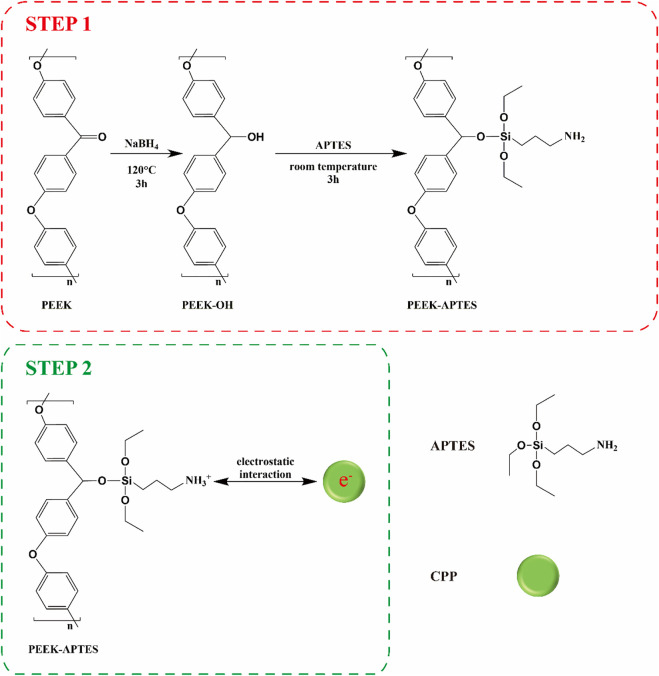
Schematic diagram illustrating the fabrication of CPP coating.

PEEK was dissected into disks (Φ: 14 mm, thickness: 1 mm). All the specimens were polished using sandpaper (400, 800, 1,200, 3,000, and 5,000 grit), ultrasonically cleaned in ethanol and ultra-pure water for 20 min sequentially, and dried at 60°C. CPP modification contained two steps (Scheme 1). The first step was to introduce amino groups onto PEEK surface to positively charge it. Briefly, PEEK specimens were immersed in a 100 mL DMSO solution containing 200 mg NaBH_4_ for 3 h at 120°C to reduce the carbonyl groups to hydroxyl groups. Next, the specimens were immersed in a solution containing 5% APTES, 5% ultrapure water, 90% absolute ethyl alcohol for 3 h at home temperature followed by dried at 60°C (Qiu et al., 2022b). The obtained smples were named as PEEK-APTES. The second step was electrostatic adsorption. The positively charged PEEK specimens were immersed in a CPP solution (10 mg/mL) and interacted with CPP overnight at room temperature (Qin et al., 2015). After dried at room temperature, the CPP-modified PEEK (PEEK-CPP) was obtained. For cell culture, the specimens were disinfected by ultraviolet light and ozone overnight.

### 3.2 Surface characterization

X-ray photoelectron spectroscopy (XPS, Kratos, AXIS Ultra DLD, United Kingdom) was employed to determine the surface chemical composition of the specimens. Scanning electron microscopy (SEM, FEI, Inspect F50, United States) was used to investigate the surface morphology of the specimens. Atomic force microscopy (AFM, Seiko, SPA400, Japan) was used to reconstruct the three-dimensional morphology and to measure the surface roughness. Contact angle goniometer (Dataphysics, OCA20, Germany) was carried out to measure the static water contact angle of the specimens and deionized water was used as the measuring liquid.

### 3.3 Coating degradation tests *in vitro*

Coating degradation test was carried out by immersing the PEEK-CPP samples in medium containing αMEM (89%), FBS (10%), penicillin-streptomycin (1%), and the weight change of the samples throughout the immersion was recorded to obtain the weight loss of the CPP coating. The samples were weighed before (*W*_*0*_) and after (*W*_*1*_) CPP modification, then cultured for 28 days at 37°C in the medium that was refreshed every 3 days. The samples were removed, washed, dried and weighed (*W*_*2*_) at immersion intervals (7, 14, 21 and 28 days). According to the following formula, the weight loss of the CPP coating was calculated:

$$\begin{array}{c} Weight\;loss=\frac{W_{1}-W_{0}}{W_{1}-W_{2}}\times 100\% \end{array}$$ (1)

### 3.4 Cell culturing

The MC3T3-E1 cells were cultured in α-MEM with 10% FBS and 1% penicillin/streptomycin at 37°C in an incubator. The medium was refreshed every 3 days. MC3T3-E1 cells were seeded on the PEEK samples at a density of 2 × 10^4^ cells/well. For osteogenic differentiation, after culturing for 2 days, the normal medium was replaced with an osteogenic differentiation medium containing 89% Dulbecco’s modified eagle medium, 10% fetal bovine serum, 1% penicillin/streptomycin, 50 μM ascorbic acid, and 10 mM β-glycerophosphate.

### 3.5 Cell adhesion

The samples were cultured for 4 h, then fixed with 4% paraformaldehyde for 30 min followed by rinsed three times with PBS. After stained by DAPI for 8 min and washed three times with PBS, the cell adhesion on the samples was examined using an inverted fluorescence microscope.

Cell adhesion was also evaluated using a CCK-8 kit. The samples were cultured for 4 h followed by rinsed three times with PBS. Each well received a dose of CCK-8 reagent in accordance with the manufacturer’s instructions. A microplate reader (BioTeke, China) was used to measure the optical density (OD) values at 450 nm following a 4-hour incubation at 37°C away from light.

A confocal laser scanning microscope (CLSM; Leica; Germany) was used to analyze the cell morphology. Following a 4-hour culture, the samples were permeabilized with 0.1% Triton X-100 for 10 min, fixed with 4% paraformaldehyde for 30 min, and blocked with 5% BSA for an hour at ambient temperature. A 200 μL solution of Acting-Tracker Green was added into each well. The samples were incubated for 1 h at room temperature, then washed three times in sterile PBS containing 0.1% Triton X-100. Finally, the samples were photographed by CLSM after stained in DAPI solution for 8 min and washed three times in sterile PBS.

### 3.6 Cell proliferation

To evaluate the cell growth on the samples, a CCK-8 kit was employed. Cell growth was assessed on days 1, 4, and 7 as previously described.

### 3.7 Alkaline phosphatase activity assay

ALP staining was performed to measure the ALP activity on days 7 and 14. Following the removal of the media, the samples were fixed in 4% paraformaldehyde for 30 min, stained with an NBT/BCIP ALP color development kit, and imaged under a stereomicroscope.

An alkaline phosphate activity assay kit was also used to assess the ALP activity on days 7 and 14 in accordance with the manufacturer’s instructions. The OD values at 520 nm were obtained to determine the ALP activity. For standardization, we used a BCA protein assay kit to examine the total protein concentration in accordance with the manufacturer’s instructions. The OD values were measured at 562 nm and total protein concentration was determined according to the standard curve. Then, the ALP activity was expressed as an activity unit (U/gprot).

### 3.8 Extracellular matrix mineralization assay

ARS staining was used to evaluate the mineralized nodule formation on days 21 and 28. The samples were fixed for 30 min in 4% paraformaldehyde, stained for 20 min at room temperature in 200 μL of ARS solution (2%), then washed three times in PBS. A stereomicroscope was used to photograph the calcium nodules. For quantification, the stained samples were soaked in hexadecyl pyridinium chloride solution (1 w/v%) for 2 h to dissolve the adsorbed alizarin red dye. The OD value of the hexadecyl pyridinium chloride solution was measured at 550 nm.

### 3.9 Real-time polymerase chain reaction analysis

RT-PCR analysis was used to assess the expression of osteogenic genes on days 7 and 14. An RNAsimple Total RNA Kit was used to extract the total RNA. A ReverTra Ace^®^ qPCR RT Master Mix was used to reverse-transcribe total RNA to cDNA according to the manufacturer’s instructions. The gene expressions of type I collagen (*Col1α1*), alkaline phosphatase (*Alp*), and runt-related transcription factor 2 (*Runx2*) were measured using a QPCR system (DNA Engine Opticon 2, Bio-rad, United States) and SYBR Green Realtime PCR Master Mix. The housekeeping gene *β-actin* was employed for standardization. The primers for various genes are listed in Table 1. On the basis of the cycle threshold values, the gene expressions were quantified.

**TABLE 1:** Primer pairs used in RT-PCR analysis.

Gene	Primers
*Runx2*	Forward: 5′-CCT​GAA​CTC​TGC​ACC​AAG​TCC​T-3′
	Reverse: 5′-TCA​TCT​GGC​TCA​GAT​AGG​AGG​G-3′
*ALP*	Forward: 5′-CCA​GAA​AGA​CAC​CTT​GAC​TGT​GG-3′
	Reverse: 5′-TCT​TGT​CCG​TGT​CGC​TCA​CCA​T-3′
*Col1α1*	Forward: 5′-CCT​CAG​GGT​ATT​GCT​GGA​CAA​C-3′
	Reverse: 5′-CAG​AAG​GAC​CTT​GTT​TGC​CAG​G-3′
*β-actin*	Forward: 5′-GGC​TGT​ATT​CCC​CTC​CAT​CG-3′
	Reverse: 5′-CCA​GTT​GGT​AAC​AAT​GCC​ATG​T-3′

### 3.10 Statistical analysis

IBM SPSS Statistics 22.0 was used to conduct the statistical analysis. The data was expressed as means ± standard deviations (SD) and analyzed statistically using the Student’s t-test. *p* < 0.05 was considered to be statistically significant.

## 4 Results and discussion

### 4.1 Surface characterization

**FIGURE 1:**
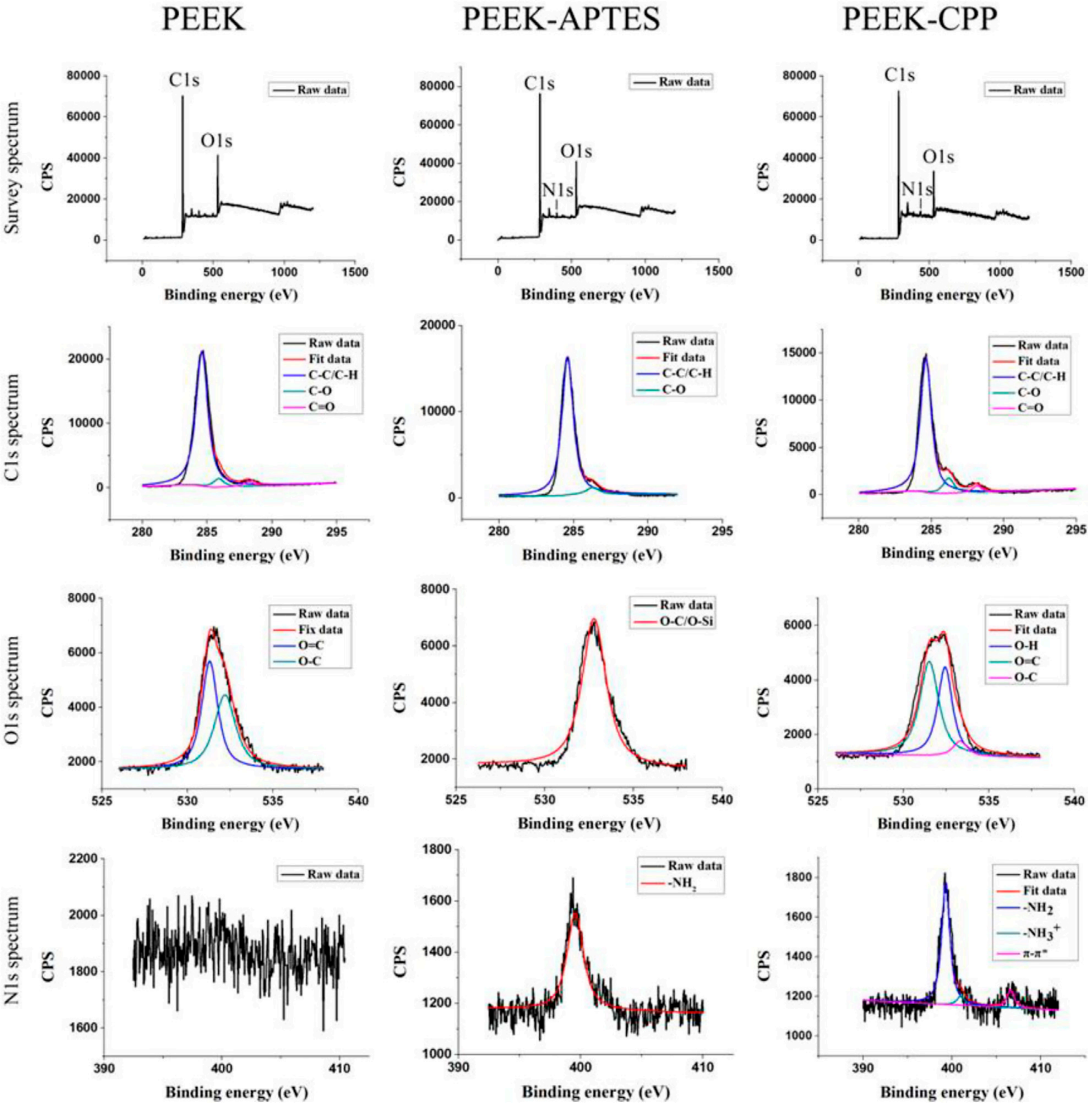
XPS survey spectra and high-resolution spectrum of C1s, O1s, and N1s.

XPS was performed on different PEEK samples to verify the successful graft of amino groups and the effective adsorption of CPP. As shown in Figure 1, C1s and O1s peaks were observed in the survey spectra of PEEK, PEEK-APTES, and PEEK-CPP, but N1s peak was only seen in the survey spectra of PEEK-APTES, and PEEK-CPP. This suggested that a substance containing N element might have been incorporated to the PEEK surface.

In the C1s spectra of PEEK, the peaks at 284.8, 286.4, and 287.1 eV correspond to aliphatic carbon (C-C/C-H), C-O and carbonyl group (C=O), respectively (Noiset et al., 1997; Biesinger et al., 2011). As the C1s spectra of PEEK-APTES shows, Carbonyl group peak was not detected, indicating that the carbonyl groups in the testing zone were reduced to hydroxyl groups. Besides, the O-C/O-Si peak at 530 eV and the -NH_2_ peak at 399.5 eV were the only peak in the O1s spectra and N1s spectra of PEEK-APTES, respectively. This is because the APTES replaced the hydroxyl groups in the testing zone. However, as shown in the C1s spectra of PEEK-CPP, the carbonyl group peak was detected again on PEEK-CPP surface. This indicated that CPP successfully adsorbed to the surface of PEEK-APTES, because the serine and glutamic acid, core structure of the CPP, contains carbonyl groups. Furthermore, the N1s spectra of PEEK-CPP shows the π-π* satellite feature, which suggests that CPP was adsorbed onto the PEEK-APTES surface because π-π* staking interaction always occurs between electron-rich molecules and electron-deficient molecules.

**FIGURE 2:**
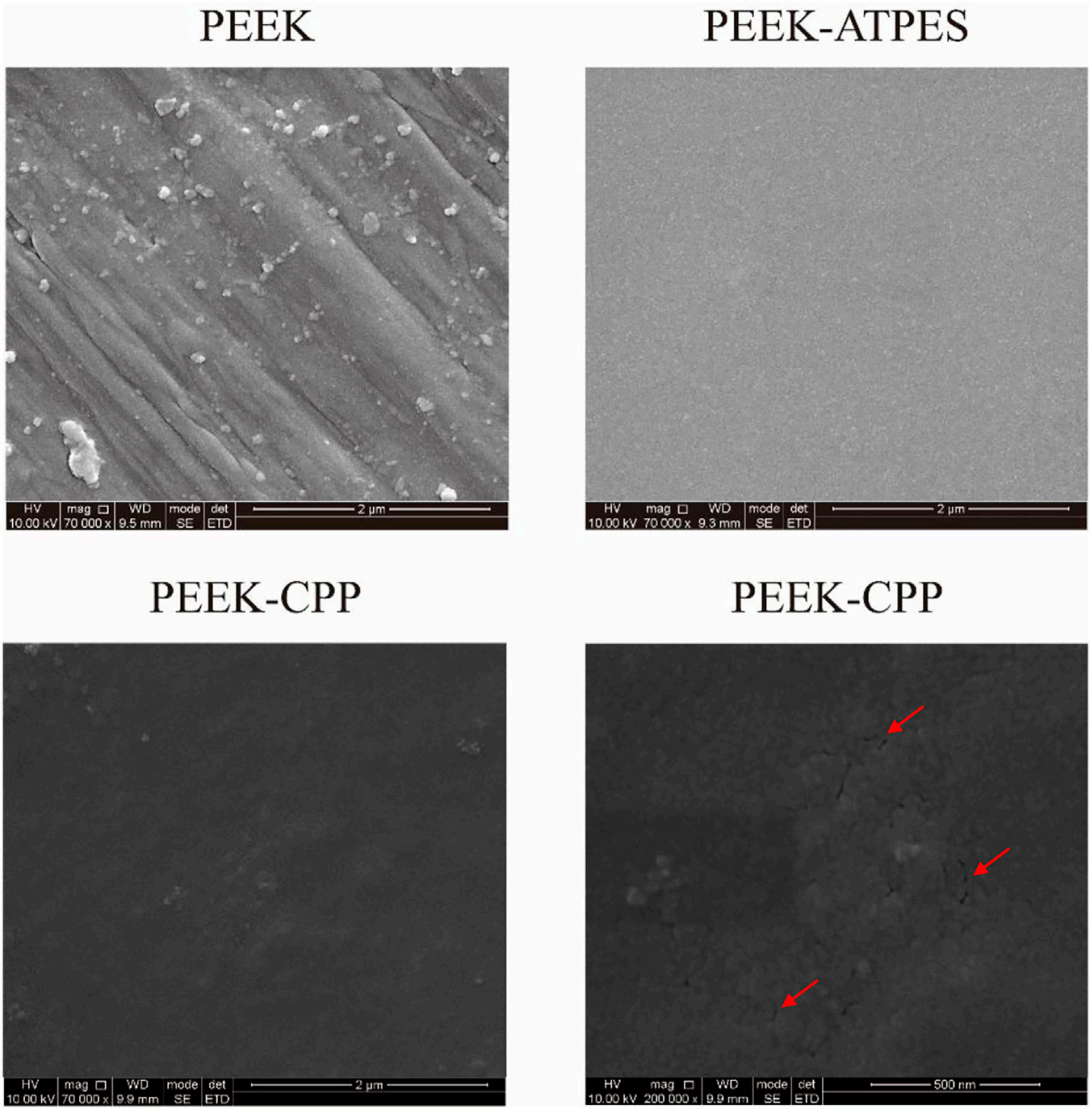
The surface morphology of different PEEK specimens by SEM observation.

**FIGURE 3:**
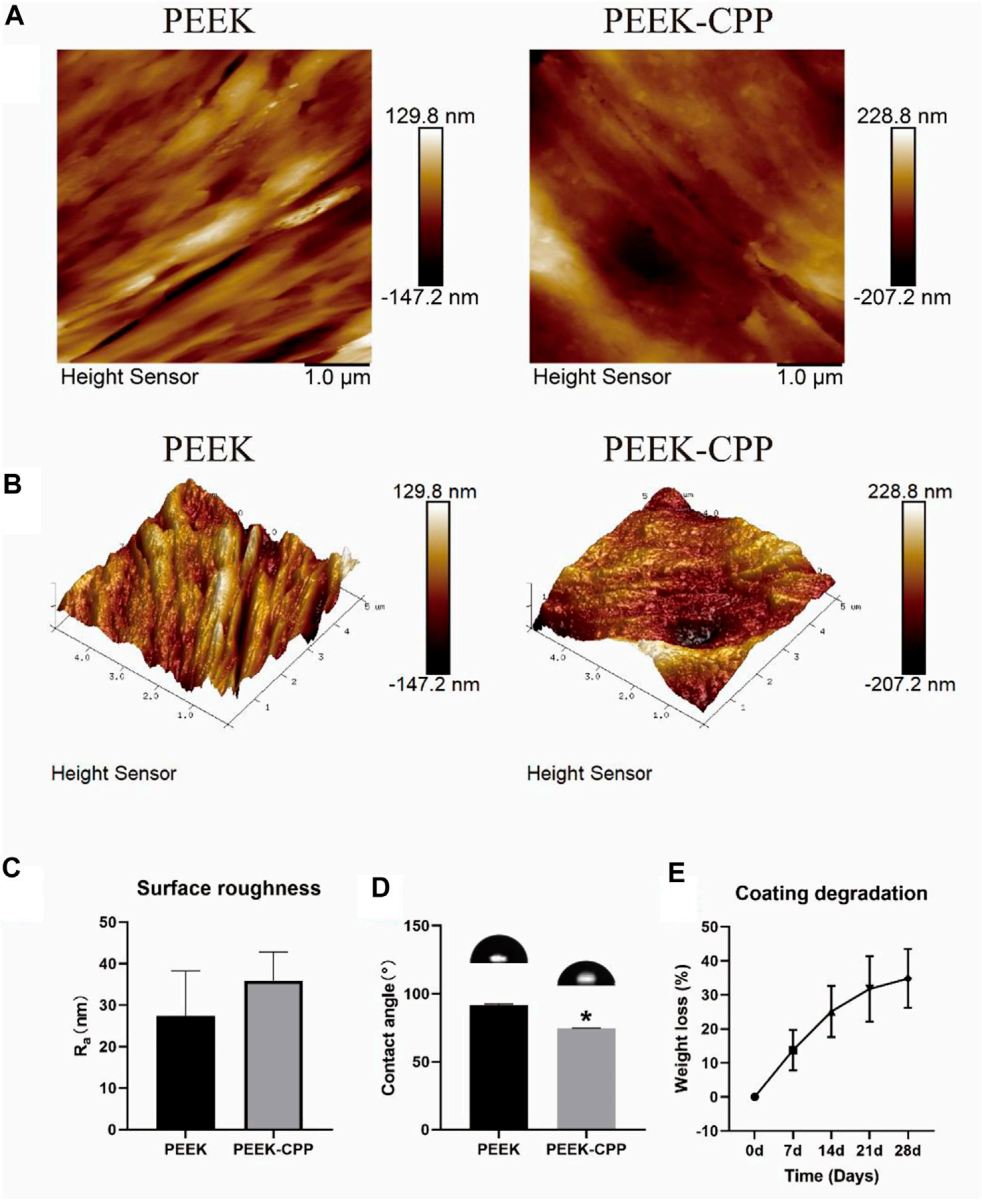
Surface characterization of different PEEK specimens and coating degradation test of PEEK-CPP specimens. **(A, B)** Two-dimensional and three-dimensional reconstructed morphologies by AFM observation. **(C)** Surface roughness (*n* = 3). **(D)** Water contact angles (*n* = 3). **(E)** Weight loss of the CPP coating (*n* = 5). * The asterisk indicates statistically significant differences compared with PEEK (*p* < 0.05).

Representative SEM images of PEEK, PEEK-APTES and PEEK-CPP are provided in Figure 2. Grooves were made on the surface of the original PEEK by polishing (at a magnification of 7 × 10^4^ times). As shown the SEM image of the PEEK-APTES sample (at a magnification of 7 × 10^4^ times), the surface was quite smooth and homogeneous after grafting APTES. However, after CPP deposition, a granular surface was shown (at a magnification of 7 × 10^4^ times) and some pores also were observed as indicated by the red arrows (at a magnification of 2 × 10^5^ times). Figures 3A,B, respectively, show the two-dimensional morphologies and three-dimensional reconstructed morphologies of PEEK and PEEK-CPP; Figure 3C depicts the surface roughness of different samples. While there was no significant difference between the PEEK’s and PEEK-CPP’s Ra values (*p* > 0.05), the deeper valleys and grooves were observed on the surface of PEEK-CPP. These pores and valleys observed on PEEK-CPP samples by the SEM and AFM could benefit the adhesion of cells (Yin et al., 2022). As shown in Figure 3D, PEEK-CPP (74.30° ± 0.26°) had a notably lower contact angle than PEEK (91.30° ± 1.10°) (*p* < 0.05). Therefore, the introduction of CPP can effectively increase the hydrophilicity of PEEK. The result was consistent with the previous research. Zengjie Fan et al. (2013) synthesized CPP modified carboxyalated graphene and found CPP modification substantially decreased the contact angle of carboxyalated graphene.

The stability of CPP the coating was determined by a coating degradation test. As shown in Figure 3E, with the extension of the soaking time, the weight loss of the CPP coating gradually increased to about 30% on day 28. This result demonstrated that the CPP coating was stably adsorbed onto PEEK surface and had a low degradation ratio in a short term. This ensured that the CPP coating could continuously improve the MC3T3-E1 cells’ functions at the early stages. Undoubtedly, the result of the stability test of the CPP coating would be more persuasive if we could also use an AFM to observe the surface morphology change of the PEEK-CPP specimens before and after the degradation test. Moreover, adding PEEK-APTES samples as a control group could help determine the precise degradation condition of the CPP layer.

### 4.2 Cell adhesion and proliferation

**FIGURE 4:**
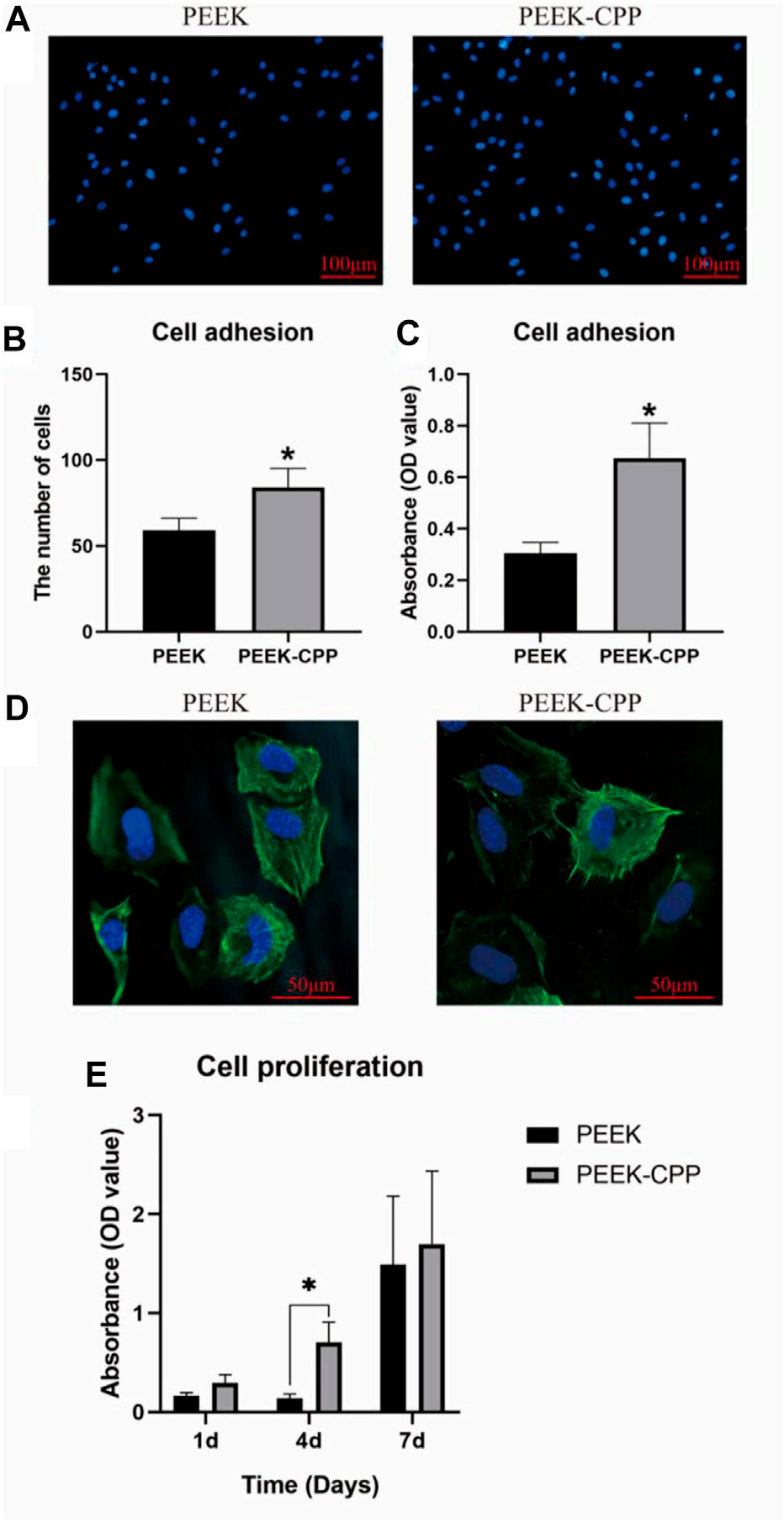
Cell adhesion and proliferation on different PEEK specimens. **(A, B)** Representative images of cell adhesion and cell count by DAPI staining (*n* = 5). **(C)** Cell adhesion tested by CCK-8 assay (*n* = 3). **(D)** Cell adhesion morphology by CLSM observation. **(E)** Cell proliferation tested by CCK-8 assay (*n* = 3). * The asterisk indicates statistically significant differences between groups (*p* < 0.05).

Cell adhesion on implants surface reflects the implants’ biocompatibility, which is the prerequisite of cell proliferation and differentiation. Figures 4A, B, respectively, illustrate the representative images and cell counts of MC3T3-E1 cells on different PEEK samples after DAPI staining. It is obvious that more cells adhered on the PEEK-CPP samples compared with the PEEK samples (*p* < 0.05). The similar result of cell adhesion was also obtained by the CCK-8 assay (Figure 4C). Furthermore, as shown in Figure 4D, the PEEK-CPP group showed more outstretched cell morphology. The cells on the surface of PEEK-CPP samples stretched better and displayed a well-developed cytoskeleton, with more pseudopods and many visible F-actin microfilaments in the cytoplasm. In contrast, the cells on the surface of PEEK samples presented less extension, fewer pseudopods and fuzzier F-actin microfilaments in the cytoplasm. Additionally, as shown in Figure 4E, PEEK-CPP group exhibited higher cell proliferation than PEEK group on days 1, 4 and 7 despite only day 4 showed the significant difference (*p* < 0.05). Moreover, the absorbances of cell proliferation on days 1 and 4 in the PEEK group were relatively low, and a dramatic increase showed between day 4 and day 7. In contrast, the absorbances of cell proliferation on days 1, 4 and 7 in PEEK-CPP group showed a uniform linear growth. These results indicated that PEEK-CPP specimens were beneficial to the proliferation of the MC3T3-E1 cells in early stages after seeding, which might enable the MC3T3-E1 cells to enter the stages of osteogenic differentiation. The findings were consistent with the previous studies. Wen Huang et al. (2022) prepared CPP-calcium chelate and found it had positive effects on the proliferation and differentiation of MC3T3-E1 cells *in vitro*. Higher cell adhesion and proliferation and better cell adhesion morphology on PEEK-CPP samples might be relevant to the deeper valleys and grooves and higher hydrophilicity on PEEK-CPP surface. Firstly, deeper valleys and pores could raise the implant material’s specific surface area and total surface area and provide additional sites and places for cell adhesion, resulting in better cell adhesion morphology (Yu et al., 2020). Secondly, higher hydrophily on PEEK-CPP could improve fibrinogen adsorption and cell adhesion on the implant surface by increasing the implant surface’s contact degree and surface tension to the wetting liquid (Conti et al., 2002). Thirdly, better cell adhesion morphology on PEEK-CPP samples could further stimulate cell proliferation. Cell proliferation is usually positively correlated with cell stretching because cells anchored to the growth matrix do not divide without stretching (Bacakova et al., 2011). However, even though we have observed the better adhesion morphology of MC3T3-E1 cells on PEEK-CPP samples by the staining of cytoskeleton, the staining of focal adhesions could help to explain why a better adhesion morphology of the cells was expressed on PEEK-CPP specimens from a mechanism perspective. Cell adhesion is modulated by adhesion receptors. For example, integrins, as an adhesion receptor, are prominently concentrated in matrix adhesions, such as focal adhesions. Vinculin, a cytoplasmic actin-binding protein, is also enriched in matrix adhesions and play a key role in the process of linking adhesion receptors to the actin cytoskeleton (Izard and Brown, 2016; Bays and DeMali, 2017). Therefore, the staining of focal adhesions to some extent is necessary.

### 4.3 *In vitro* osteogenic differentiation

**FIGURE 5:**
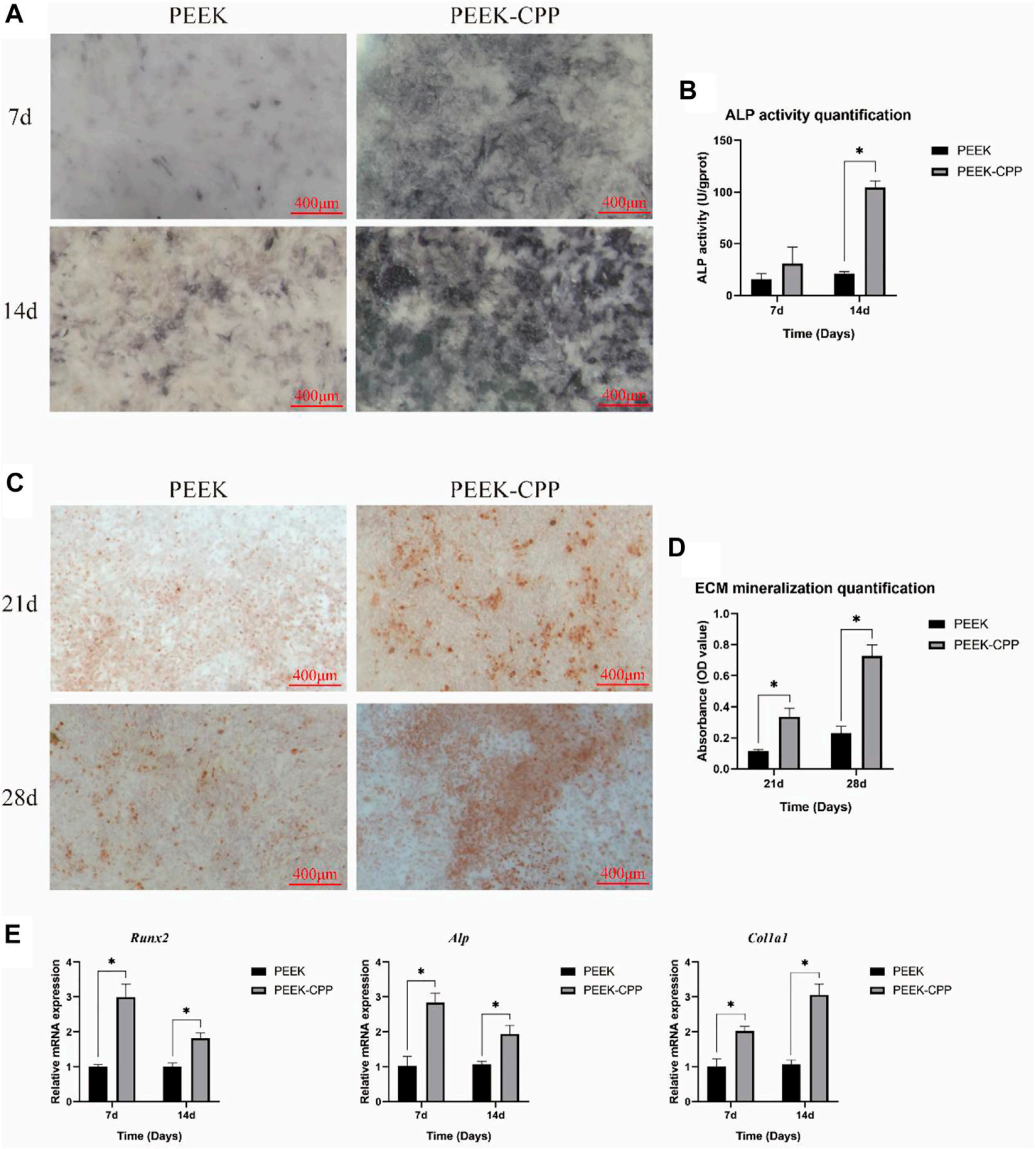
*In vitro* osteogenic differentiation. **(A, B)** Representative staining and quantification of ALP activity (*n* = 3). **(C, D)** Representative ARS staining and quantification of ECM mineralization (*n* = 3). **(E)** Expressions of osteogenic genes (*n* = 3). * The asterisk indicates statistically significant differences between groups (*p* < 0.05).

Cell osteogenic differentiation on implant surface is critical for bone rebuilding. ALP activity and ECM mineralization are the key indicators of osteogenic differentiation in the early and late stages, respectively. Figures 5A, B demonstrate the ALP activity staining and quantification of MC3T3-E1 cells on different samples after culturation for 7 and 14 days. As shown in Figure 5A, the ALP staining was deeper on PEEK-CPP samples both on days 7 and 14. Similarly, the ALP activity quantification on PEEK-CPP samples was also significantly higher than that on PEEK samples on day 14 (*p* < 0.05). Figures 5C,D illustrate the ECM mineralization. On both days 21 and 28, PEEK-CPP groups displayed more mineralized bone nodular structure and substantially higher ECM mineralization quantification (*p* < 0.05). The expressions of osteogenic genes are shown in Figure 5E. On days 7 and 14, the gene expression of *Alp* of MC3T3-E1 cells in the PEEK-CPP group was remarkably higher than that in the PEEK group (*p* < 0.05), similar to the results of ALP activity staining and quantification. The expression of *Runx2*, an osteoblast-specific transcription factor, also was higher in PEEK-CPP group than in PEEK group (*p* < 0.05). In comparison with PEEK group, the expression level of *Col1α1* (a marker related to production of ECM) was considerably upregulated in PEEK-CPP group (*p* < 0.05). These results suggested that the CPP layer on PEEK-CPP specimens could enhance the osteogenic differentiation of MC3T3-E1 cells *in vitro*. Firstly, the CPP coating indirectly enhanced the osteogenic differentiation through improving the cell adhesion and the cell proliferation on the PEEK-CPP specimens. There is research indicating that cells express functions only when they are well stretched (Reynolds, 1998). Secondly, the enhanced osteogenic differentiation may be associated to the CPP’s ability of chelating Ca^2+^. As is well known, CPP is a Ca^2+^ chelator. The phosphate groups in CPP can form polar and acidic domains which are beneficial to bond divalent Ca^2+^ Fan et al., 2013. These bonding Ca^2+^ will supply a possible source for the deposition of calcium phosphate. In an *in vitro* experiment, Alaa E. Dawood et al. found casein phosphopeptide-amorphous calcium phosphate composite (CPP-ACP) was biocompatible with the potential to induce osteoblastic differentiation and calcification/mineralization, which was related to the CPP’s ability of stabilizing bioavailable Ca^2+^ and phosphate ions and inducing uptake of Ca^2+^ Dawood et al., 2018. The cluster sequence of Ser(P)-Ser(P)-Ser-(P)-Glu-Glu in CPP can stabilize nanoclusters of ACP, resulting in increased calcium phosphate levels and decreased diffusion of free Ca^2+^ Reynolds, 1998. Additionally, CPP’s numerous phosphoserine groups can be employed as nucleation and growth sites of calcium phosphate, which is favorable to the ECM mineralization on PEEK-CPP substrates (Fan et al., 2013). There was a study indicating that CPP-functionalization could mediate formation of hydroxyapatite on the surface of substrates in simulate body fluid (SBF). Furthermore, some studies showed that CPP could facilitate the transcellular transport of Ca^2+^ Li et al., 2021. Liu et al. (2018) found that CPP feeding substantially increased the femur index, serum Ca^2+^ and serum osteocalcin levels, and femoral calcium content in rats. Moreover, in a study on the osteogenic ability of CPP-calcium chelate, it was proven that there were 321 differently expressed genes found in MC3T3-E1 cells and CPP might stimulate the osteogenic differentiation through AMPK signaling pathway, PI3K-Akt signaling pathway, MAPK signaling pathway, and Wnt signaling pathway, and the expression of NOTUM, WIF1, LRP4, APOD, OGN, and IGF1 (Huang et al., 2022).

*In vitro*, PEEK-CPP demonstrated higher cell adhesion, improved spreading cell morphology, increased cell proliferation, ALP activity and ECM mineralization and upregulated expressions of osteogenic genes. These findings suggested that PEEK-CPP exhibited better osteoinductive ability than PEEK *in vitro*. Even though we do have observed the improved biocompatibility and osteoinductivity on the surface of PEEK-CPP samples, there still are shortcomings in our study. Firstly, it would be better to determine the effect of CPP layer on improving the biocompatibility and osteoinductivity of PEEK-CPP samples if the biocompatibility and osteoinductivity of PEEK-APTES samples were tested. Secondly, the experiments evaluating the expression of osteogenesis related proteins *in vitro* and the osseointegration *in vivo* need to be carried out to further confirm the osteoinductive ability of PEEK-CPP. Moreover, in order to make sure the long-term stability of PEEK-CPP implants under functional conditions, their mechanical characteristics should be tested. Finally, improving the degradation rate of CPP layer to obtain a long-term osteogenic ability may be an interesting and potential research direction in the future.

## 5 Conclusion

In this study, a CPP layer was electrostatically adsorbed onto positively charged PEEK through a two-step strategy. *In vitro*, the CPP-modified PEEK demonstrated osteogenic ability. This simple CPP modification has great potential in the bio-functionalization of the PEEK implants study. However, for dental applications, more molecular biology and *in vivo* research and mechanical property tests should be conducted.

## Data Availability

The raw data supporting the conclusion of this article will be made available by the authors, without undue reservation.
